# Initiation of antidepressant use among refugee and Swedish-born youth after diagnosis of a common mental disorder: findings from the REMAIN study

**DOI:** 10.1007/s00127-020-01951-4

**Published:** 2020-09-10

**Authors:** Heidi Taipale, Thomas Niederkrotenthaler, Magnus Helgesson, Marit Sijbrandij, Lisa Berg, Antti Tanskanen, Ellenor Mittendorfer-Rutz

**Affiliations:** 1 Division of Insurance Medicine, Department of Clinical Neuroscience, Karolinska Institutet, 171 77 Stockholm, Sweden; 2 Niuvanniemi Hospital, Kuopio, Finland; 3 Unit Suicide Research and Mental Health Promotion, Department of Social and Preventive Medicine, Center for Public Health, Medical University of Vienna, Vienna, Austria; 4 Department of Clinical, Neuro- and Developmental Psychology, World Health Organization Collaborating Centre for Research and Dissemination of Psychological Interventions, Vrije Universiteit, Amsterdam, The Netherlands; 5 Department of Public Health Sciences, Stockholm University, Stockholm, Sweden

**Keywords:** Antidepressant, Refugee, Pharmacotherapy, Depression, Anxiety disorders

## Abstract

**Purpose:**

The objective of this study was to compare the initiation and type of antidepressant use between refugees and matched Swedish-born youth after a diagnosis of a common mental disorder (CMD) and assess sociodemographic and clinical factors associated with the initiation.

**Methods:**

The study cohort included youth aged 16–25 years, with an incident diagnosis of CMD based on specialized health care registers in Sweden 2006–2016, without prior antidepressant use during 1 year. One Swedish-born person was matched for each identified refugee youth (*N* = 3936 in both groups). Initiation of antidepressant use and factors associated with the initiation, were investigated with logistic regression yielding Odds ratios, OR, and 95% Confidence Intervals, CI.

**Results:**

Refugees were less likely to initiate antidepressant use compared with Swedish-born (40.5% vs. 59.6%, adjusted OR 0.43, 95% CI 0.39–0.48). Selective serotonin reuptake inhibitors (SSRIs) were less frequently initiated for refugees than Swedish-born (71.2% vs. 81.3% of initiations, *p* < 0.0001). Sertraline was the most commonly initiated antidepressant both for refugees (34.3%) and Swedish-born individuals (40.3%). Among refugees, factors associated with increased odds of antidepressant initiation were previous use of anxiolytics or hypnotics, previous sickness absence of < 90 days, cancer and older age (OR range 1.07–2.72), and less than 5 years duration of residency in Sweden was associated with decreased odds (OR 0.76, 95% CI 0.63–0.92).

**Conclusion:**

Young refugees with a CMD seem to initiate antidepressants in general and those most effective considerably less often than their Swedish-born counterparts.

**Electronic supplementary material:**

The online version of this article (10.1007/s00127-020-01951-4) contains supplementary material, which is available to authorized users.

## Introduction

Mental disorders are frequent among young refugees resettled in European countries [1–3]. They have an increased risk for common mental disorders (CMDs), namely depression and anxiety disorders, including post-traumatic stress disorder (PTSD), which is significantly more prevalent than in youth born in the respective host country [4]. However, also depression and anxiety disorders are common and prevalence estimates vary widely between studies [3]. Among refugees, children and adolescents are often considered as the most vulnerable group as they are experiencing the stressors of forced migration during their formative years. Refugee youth may be at a higher risk for mental ill-health due to adverse experiences, such as violent and traumatic events before and during the migration process [5]. In addition, they may face problems when adapting to the new host country, such as discrimination, bullying, stressful family circumstances, and distressing legal processes of gaining refugee status and permanent residency permit [5].

However, a higher risk of mental disorders may not always translate into a higher utilization of mental health care and treatment. Medication use for mental disorders is often reported to be less frequent in adult refugee and migrant populations in comparison to persons born in the host country in Nordic countries [6–8]. This may be caused by barriers in accessing health care in general and individual-level factors related to socio-cultural perceptions and attitudes, but also practical problems such as language difficulties when communicating with health-care professionals [9]. A previous Swedish study found that newly resettled refugee adults were less likely to be dispensed psychotropic drugs than Swedish-born, but the likelihood of dispensing increased with longer duration of residence [6]. This indicates that particularly newly resettled refugees may have barriers in the access to mental health care. A Danish study compared the initiation of antidepressant use after inpatient care due to moderate to severe depression between Danish-born and non-Western immigrants and found that immigrants were less likely to initiate antidepressant treatment, although the significance of the association was attenuated when adjusted for income [7]. Another previous study assessed risk factors for not initiating antidepressant use after general practitioner recommendation and found that non-Western immigrants had higher odds for declining the treatment than other patients [10].

In general, previous studies indicate that health care utilization is lower in migrants than in the majority population of the host country, particularly specialised health care due to mental disorders in refugees [11]. None of the previous studies has, however, assessed whether antidepressant initiation differs between refugee youth and youth born in the host country after a diagnosis of a common mental disorder. Although differences in the response to pharmacotherapy have been demonstrated for pediatric and adult depression, such as less efficacy and higher likelihood for adverse effects for children and adolescents [12], reviews and guidelines for youth depression and anxiety disorders generally recommend antidepressant use, in addition to cognitive behavioural therapy [13–15]. For PTSD, clinical care guidelines emphasize psychotherapy and even advice against pharmacological treatments for young people aged less than 18 years [16] but pharmacotherapies still may be used, especially if psychotherapy options are not readily available. A recent systematic review and meta-analysis reported that selective serotonin reuptake inhibitors (SSRIs) are efficacious for children and adolescent depression and anxiety disorders, and noticed a considerable lack of evidence of these medications for treatment of PTSD [17]. A previous meta-analyses assessing specific drugs reported that only fluoxetine was significantly more effective than placebo for depression among children and adolescents [18]. Despite lack of evidence on efficacy and safety of other psychopharmacotherapies, they may be prescribed as an attempt to avoid adverse effects of antidepressants, e.g., risk of suicidality potentially associated with any antidepressant use in adolescents [14].

There is a lack of scientific knowledge on the initiation of pharmacotherapy in refugee youth with common mental disorders. Moreover, scientific information is lacking which type of pharmacotherapy is initiated and which factors influence the initiation. Here, it is important to investigate the importance of a range of factors, i.e., not only clinical, but also sociodemographic characteristics, such as duration of residency in the host country. This information is crucial to design person-based and culturally sensitive pharmacological treatment for refugee youth. The objective of our study was to compare the initiation of antidepressant use between refugee and matched Swedish-born youth after diagnoses of CMD and assess sociodemographic and clinical factors associated with initiation of use. Further objectives were to assess whether the type of antidepressant and frequency of initiation of psychopharmacotherapy other than antidepressants differed between refugees and Swedish-born.

## Methods

The study base included all persons aged 16–25 years who had a main diagnosis of CMD in the National Patient Register (NPR; in- or specialized outpatient care) during 2006–2016 in Sweden. CMD was defined as a major depressive disorder [with International Classification of Diseases (ICD) version 10 codes F32, F33], anxiety disorder (excluding PTSD, F40–F43 excluding F43.1, short as “anxiety disorder”) and post-traumatic stress disorder (PTSD, F43.1). Exclusion criteria were a previous occurrence of any of the following ICD-10 diagnoses in the NPR at any time before the first CMD diagnoses: psychotic disorders (ICD-10: F20–F29), manic episode (F30) or bipolar disorder (F31).

Data linkage between several population-based registers was conducted via the unique (de-identified) Swedish personal identity number. Sociodemographic, labor market and educational data was derived from the Longitudinal Integration Database for Health Insurance and Labor Market Studies (LISA) held by Statistics Sweden. Refugee status was derived from STATIV (a longitudinal database for integration studies) containing migration-related information, whereas the National Patient Register was utilized for deriving inpatient care (since 1997) and outpatient care (since 2001) data on comorbid conditions and hospital care periods. The Prescribed Drug Register (PDR) provided information on drug use (since July 2005) and the Cause of Death Register all deaths of Swedish residents (since 1952).

### Refugees

We included persons who were residing in Sweden during the year prior to the CMD diagnoses with refugee status i.e., having received a residence permit in Sweden as a refugee, or as an individual who has been granted residence permit due to “in need of protection” or “humanitarian grounds” or through “family reunification”.

### Matching

To derive a matched cohort of comparison persons, we matched each refugee with one control diagnosed with the same CMD type (depression/anxiety disorder/PTSD), but who had been born in Sweden with both parents born in Sweden. Matching criteria were the calendar year of CMD diagnoses, type of CMD (depression/anxiety disorder/PTSD), age (± 4 years), gender and place of residence (large city, medium-sized municipality, small municipality). The matching resulted in *N* = 6862 refugees with *N* = 6742 Swedish-born controls as comparison persons (lack of comparison persons for refugees was due to lack of PTSD cases in the Swedish-born youth cohort where matching was conducted).

### Exposure

The main exposure assessed in this study were antidepressants, defined as Anatomical-Therapeutic-Chemical (ATC) classification codes N06A. Antidepressants were categorized as selective serotonin reuptake inhibitors (SSRIs) and other antidepressants, and by assessing use of the most common drug substances. To investigate possible differences in treatment choices, use of anxiolytics (ATC code N05B), sedative-hypnotics (N05C), mood stabilizers (carbamazepine N03AF01, valproic acid N03AG01, lamotrigine N03AX09, lithium N05AN01) and antipsychotics (N05A excl. lithium) were assessed. These were considered as secondary drug category and named as “other psychopharmacotherapy”. Drug use was modelled with the PRE2DUP method [19]. This method constructs drug use periods, i.e., when drug use started and ended from drug dispensing recorded in the Prescribed Drug Register data. Each ATC code for each person is modelled separately. The method is based on calculation of sliding averages of daily dose in defined daily dose (DDD) according to the individual drug use patterns. It considers hospitalizations (when drugs are provided by the caring unit and not recorded in the register), stockpiling of drugs and changing doses. The method is based on ATC-codes and parameters related to the Nordic Article Number (vnr-number, i.e., package identifier), which restricts joining of purchases according to clinically relevant minimum and maximum doses.

### Study design

As initiation of use was the main focus, we excluded persons who had less than 1 year of follow-up after the CMD diagnoses to ensure that they had proper time to initiate the use (Supplementary Fig. 1). The analyses were restricted to new users who were identified with a 1-year washout period for antidepressant use (main exposure) or for other psychopharmacotherapy (secondary exposure and analyses). As it is likely that persons have been treated in primary care before their first contact to specialized health care, new initiations were considered 3 months before and 1 year after their first recorded CMD diagnoses (referred as assessment period for initiations). The washout period was from 15 to 3 months before CMD diagnoses, and analyses were conducted separately for antidepressant use and other psychopharmacotherapy use. After these exclusions, the matching was retained in both cohorts (i.e., refugees without a comparison person were excluded and vice versa), resulting in *N* = 3936 refugees and *N* = 3936 Swedish-born in the antidepressant cohort and *N* = 4506 refugees and *N* = 4506 Swedish-born individuals in the other psychopharmacotherapy cohort.

### Covariates

A range of sociodemographic and clinical covariates were included. Psychiatric comorbidities included previous diagnoses of other CMD type (than the current one and recorded as side diagnoses), substance abuse disorders ((F10–16, F18–19; or use of drugs for addictive disorders, including disulfiram (ATC N07BB01), acamprosate (N07BB03), naltrexone (N07BB04), nalmefene (N07BB05), sublingual buprenorphine (N07BC01/N07BC51) or methadone (N07BC02)), eating disorders (ICD-10 F50), attention-deficit hyperactivity (ADHD) or other Hyperkinetic disorders (F90; or use of ATC code N06BA), other mental and behavior disorder (by excluding the above mentioned disorders) and previous suicide attempt (ICD codes X60-X84 and Y10-Y34). We also assessed whether individuals had previous psychiatric hospital admission (ICD-10 F00-99) or non-psychiatric hospital admission (other than F00-99 as main diagnoses). Somatic comorbidities included cancer (C00-D48), asthma (J45-46), other respiratory disease (J00-99, excluding J45-46), musculoskeletal disease (M00-M99), and all other somatic diseases (ICD-10 A00-N99, excluding above mentioned diseases). Clinical covariates were assessed during 3 years before the assessment period for antidepressant use began.

Sociodemographic factors were assessed at the end of the year preceding the diagnoses of CMD, and included educational level (low, medium and high) and family situation (married/cohabiting, living without children; married/cohabiting, living with children; single, living without children; single, living with children; missing). Characteristics covering work disability comprised sickness absence (0, ≤ 90 and > 90 net days during the year preceding the index CMD diagnosis) and disability pension (DP) at the time of the diagnoses of CMD. Migration-related factors for refugees included duration of formal residency in Sweden (≤ 5 years, 6–10 years, > 10 years) and country of birth (Former Yugoslavia, Somalia, other Africa (other than Somalia), Iraq, Iran, Syria, Afghanistan, other Asia (other than previously mentioned), Chile/other South America, and other/unknown. Other medication use was assessed during 180 days before the CMD diagnoses, including use of anxiolytics (ATC-code N05B), hypnotics (N05C), opioids (N02A) and antipsychotics (N05A excl. N05AN01). For initiated antidepressants, we calculated the dispensing lag (for those who initiated the use) as time lapse between prescription date and dispensing date of the initiated antidepressants, and categorized as < 7 days vs. ≥ 7 days (indicating longer consideration time, whether to purchase the prescribed medication or not).

### Analyses

We investigated the proportion of those who initiated antidepressant use during the assessment period, i.e., within 3 months before and until 1 year after CMD diagnoses among refugee and Swedish-born youth, and whether the type of initiated medications differed. Main exposure was antidepressant initiation and the secondary category was other psychopharmacotherapy, including anxiolytics, sedative-hypnotics, mood stabilizers and antipsychotics. Descriptive statistics regarding differences of various characteristics between refugees and Swedish-born as well as between initiators and non-initiators are presented with means, standard deviations (SD), and percentages for categorical covariates. Chi squared test was used to show differences in clinical and sociodemographic factors of refugee and Swedish-born youth (Supplementary Table 1).

**Fig. 1:**
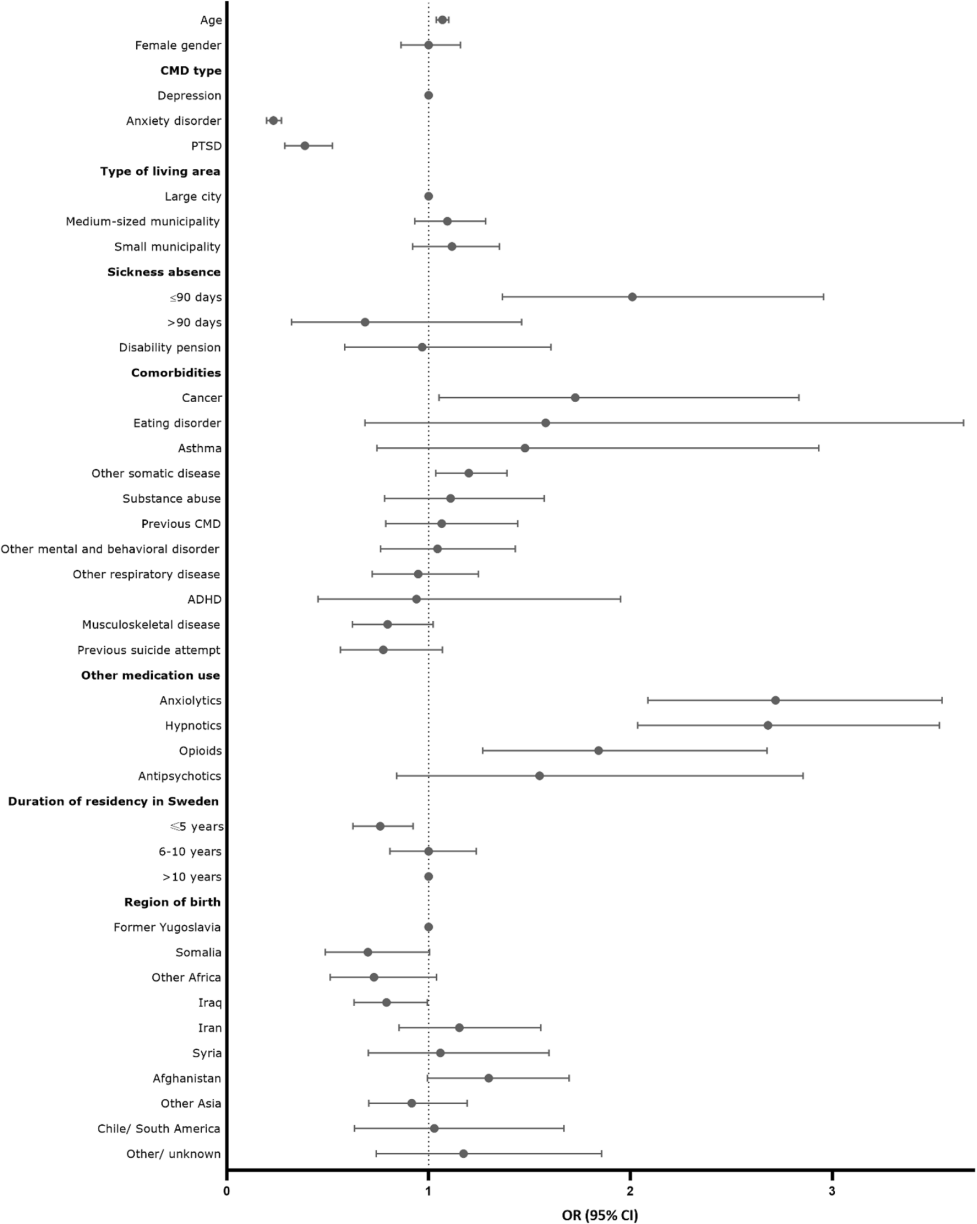
Adjusted* Odds Ratios and 95% Confidence Intervals of factors associated with antidepressant initiation among refugees 16–25 years of age with common mental disorders, CMD, resettled in Sweden. Adjusted for all factors shown. *PTSD* post-traumatic stress disorder, *ADHD* attention deficit hyperactivity disorder

**Fig. 2:**
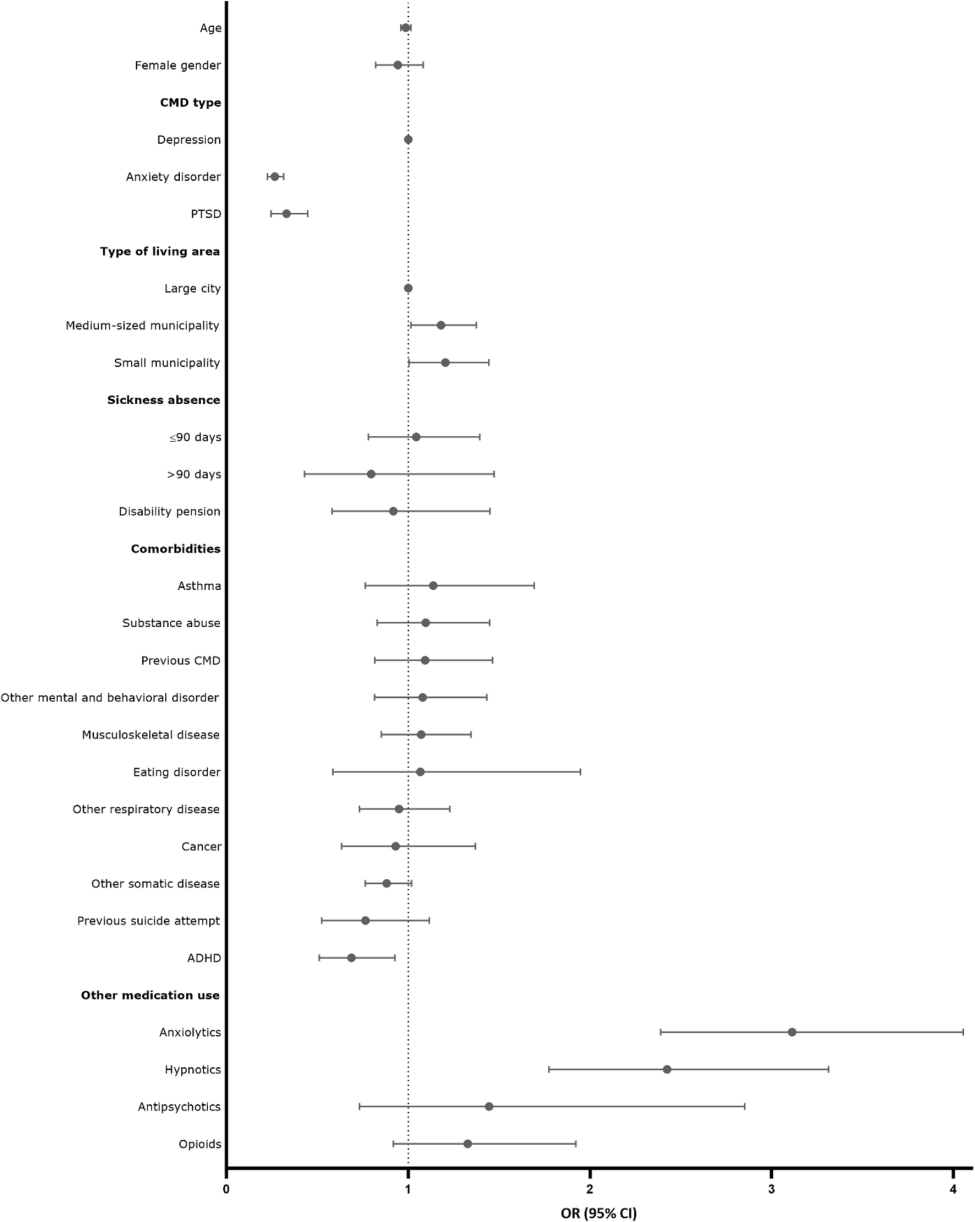
Adjusted* Odds Ratios (ORs) and 95% Confidence Intervals (CI) of factors associated with antidepressant initiation among Swedish-born youth 16–25 years of age with common mental disorders, CMD (2006–2016) in Sweden. Adjusted for all factors shown. *PTSD* post-traumatic stress disorder, *ADHD* attention deficit hyperactivity disorder

Mutually adjusted logistic regression analyses were further used to calculate the difference in initiation and to identify factors associated with initiation of antidepressant use (vs. not initiating). These factors are described in the covariates section and the final set of covariates is presented in Figs. 1, 2. Refugee and Swedish-born youth were analysed in the same model to determine whether refugee status was associated with antidepressant initiation after adjusting for potential confounding factors (all factors in Fig. 2). To further assess potential differences in the associated factors, stratified analyses within refugees and Swedish-born were conducted to explore refugee-specific factors of initiation of use (such as country of birth and duration of formal residency in Sweden). The results are reported as odds ratios (ORs) with 95% confidence intervals (CIs).

## Results

Due to the matched design, refugee and Swedish-born youth had both genders approximately equally represented (50.9% of women in both cohorts), with the mean ages being 20.5 years in both cohorts. Anxiety disorders were the most common CMD diagnoses (66.2% in both cohorts), followed by depression (27.5%) and PTSD (6.3%) (Supplementary Table 1).

Initiation of antidepressant use was less common among refugees compared with Swedish-born (40.5% vs. 59.6%, Table 1). The adjusted OR for initiating antidepressant use comparing refugees to Swedish-born was OR 0.43 (95% CI 0.39–0.48) (analyses fully adjusted for all factors in Fig. 2). This was noticed in all subgroups of CMDs, depression OR 0.47 (95% CI 0.39–0.58), anxiety disorders OR 0.42 (95% CI 0.37–0.47) and PTSD OR 0.42 (95% CI 0.28–0.63).

**Table 1:** Characteristics of initiators and non-initiators of antidepressants in a group of refugees and matched Swedish born individuals 16–25 years of age with common mental disorders, CMD (2006–2016, *N* = 3936 refugees and *N* = 3936 Swedish-born) in Sweden

	Non-initiators *N* = 3978	Initiators *N* = 3894	*p*-value
Female gender	51.4 (2044)	50.5 (1928)	0.4273
Mean age (SD)	20.3 (2.7)	20.6 (2.6)	
Immigration status			
Refugee	59.4 (2361)	40.5 (1575)	< 0.0001
Swedish-born	40.7 (1617)	59.6 (2319)	
CMD type			
Depression	15.4 (613)	39.8 (1549)	< 0.0001
PTSD	6.3 (251)	6.3 (245)	
Other anxiety disorder	78.3 (3114)	53.9 (2100)	
Type of living area^b^			
Small municipality	20.7 (822)	21.8 (848)	0.4383
Medium-sized municipality	36.7 (1458)	36.6 (1424)	
Large city	42.7 (1698)	41.7 (1622)	
Sickness absence			
≤ 90 days	3.7 (148)	6.7 (261)	< 0.0001
> 90 days	0.9 (37)	1.2 (46)	
Disability pension	2.0 (80)	2.3 (88)	0.4450
Previous diagnoses (during 3 years before index CMD diagnosis)			
Any mental and behavioral disorder^a^	16.2 (643)	19.4 (754)	0.0002
Previous CMD	5.6 (223)	7.2 (279)	0.0046
Substance abuse	5.0 (200)	6.4 (249)	0.0089
Eating disorder	0.9 (36)	1.6 (64)	0.0034
ADHD/other hyperactivity disorder	3.4 (136)	3.7 (142)	0.584
Other mental and behavioral disorder	6.7 (268)	8.7 (339)	0.0011
Previous suicide attempt	5.0 (198)	4.1 (160)	0.0644
Previous psychiatric hospital admission	4.5 (178)	5.5 (214)	0.0373
Previous non-psychiatric hospitalization	22.8 (905)	21.7 (846)	0.2745
Cancer	2.2 (87)	3.1 (118)	0.0188
Asthma	1.7 (67)	2.7 (103)	0.0034
Other respiratory disease	7.4 (293)	7.6 (297)	0.6594
Musculoskeletal disorder	9.8 (388)	10.0 (390)	0.6972
Other somatic disease	37.4 (1489)	38.9 (1516)	0.1706
Previous drug use (< 6 months)			
Anxiolytics	4.8 (189)	14.3 (556)	< 0.0001
Hypnotics	4.0 (160)	11.6 (452)	< 0.0001
Opioids	2.8 (112)	5.2 (201)	< 0.0001
Antipsychotics	0.9 (36)	2.0 (77)	< 0.0001

*CMD* common mental health disorder, *PTSD* post-traumatic stress disorder, *ADHD* attention deficit hyperactivity disorder, *p*-value derived from Chi squared-test

^a^Any mental and behavioural disorder defined as ICD-10 F00-F99

^b^Measured in the year preceding the index CMD diagnosis

In the descriptive analyses, initiation of antidepressant use was more common among those with depression, compared with anxiety disorders and PTSD, those with any previous mental and behavioral disorder and those previously using anxiolytics, hypnotics, opioids, or antipsychotics (Table 1). Among those having their first CMD diagnoses from inpatient care (*N* = 970, 59.4% refugees), 39.4% of refugees and 64.7% of Swedish-born initiated antidepressant use (*p* < 0.0001), after having a median of 4 days (IQR 2–9) stay in inpatient care (data not shown).

The class of SSRIs was less frequently initiated for refugees (71.2% of initiators) then for Swedish-born (81.3%) (Table 2). The five most commonly initiated antidepressants also differed between refugees and Swedish-born. Sertraline was the most commonly initiated antidepressant both for refugees (34.3%) and Swedish-born (40.3%), mirtazapine was second most common for refugees (21.7% vs. 11.1% for Swedish-born), whereas fluoxetine was second most common for Swedish-born (15.0%) and forth most common for refugees (12.3%). Refugees had more often ≥ 7 days lag between prescription and dispensing of antidepressants (16.8%) than Swedish-born (8.6%).

**Table 2:** Initiations of antidepressants (AD) and other psychopharmacotherapies in a group of refugees and type and year of CMD, age, gender and place of residence matched Swedish born individuals 16–25 years of age with common mental disorders, CMD (2006–2016, *N* = 3936 in each group) in Sweden

	Swedish-born % (*N*)	Refugees % (*N*)	*p*-value
**Any antidepressant**	**100.0% (2319)**	**100.0% (1575)**	
Antidepressant categories			
SSRI	81.3 (1885)	71.2 (1121)	< 0.0001
Other AD	18.7 (434)	28.8 (454)	
Five most common ADs			
Sertraline	40.3 (935)	34.3 (540)	0.0001
Mirtazapine	11.1 (258)	21.7 (341)	< 0.0001
Citalopram	14.6 (339)	13.5 (5.5)	0.3365
Fluoxetine	15.0 (348)	12.3 (194)	0.0174
Escitalopram	9.5 (220)	9.7 (3.9)	0.8644
Other AD	8.3 (193)	7.1 (112)	0.1673
> 1 AD	1.1 (26)	1.5 (23)	0.3514
Initiation by CMD type			
Depression	63.5 (389)	44.7 (692)	< 0.0001
PTSD	58.2 (146)	41.6 (102)	0.0002
Other anxiety disorder	58.6 (1826)	37.2 (781)	< 0.0001
Dispensing lag			
< 7 days	91.4 (2119)	83.2 (1311)	< 0.0001
≥ 7 days	8.6 (200)	16.8 (264)	
**Other psychopharmaco-therapy**	**100.0 (2434)**	**100.0 (2028)**	
Categories			
Anxiolytic	60.0 (1451)	52.3 (1057)	< 0.0001
Hypnotic	32.8 (793)	38.4 (776)	
Antipsychotic	6.0 (144)	8.8 (4.0)	
Mood stabilizer	1.2 (30)	0.6 (12)	
Five most common specific drugs			
Hydroxyzine	40.5 (986)	34.6 (701)	< 0.0001
Propiomazine	11.2 (272)	17.1 (347)	< 0.0001
Zopiclone	8.9 (216)	10.8 (218)	0.0353
Oxazepam	6.0 (146)	4.7 (96)	0.0633
Melatonin	5.9 (143)	3.6 (72)	0.0003
> 1 drugs	14.0 (341)	15.8 (321)	0.0888
Other	13.6 (330)	13.5 (273)	0.9253
Initiation by type of CMD			
Depression	53.3 (655)	47.2 (686)	0.0015
PTSD	55.3 (126)	46.2 (146)	0.037
Other anxiety disorder	54.9 (1697)	44.4 (1196)	< 0.0001

*CMD* common mental health disorder, *PTSD* post-traumatic stress disorder, *SSRI* selective serotonin reuptake inhibitor; *p*-value derived from Chi squared-test

Other psychopharmacotherapy was also initiated less often among refugee youth (51.5%) than Swedish-born (61.8%). Anxiolytics were less common (52.3% vs. 60.0%) and hypnotics more common (38.4% vs. 32.8%) for refugees than for Swedish-born (Table 2). The five most common specific drugs also differed although the rank order in frequency was the same for both cohorts: hydroxyzine was less common (34.6% vs. 40.5%) and propiomazine more common (17.1% vs. 11.2%) in refugees than in Swedish-born.

The factors associated with antidepressant initiation were separately assessed among refugees and Swedish-born (Table 3, Figs. 1, 2, Supplementary Table 2). Among refugees, factors associated with increased odds of antidepressant initiation were previous use of anxiolytics (OR 2.72, 95% CI 2.09–3.54), hypnotics (OR 2.68, 95% CI 2.04–3.53) and opioids (OR 1.84, 95% CI 1.27–2.68), previous sickness absence of < 90 days (OR 2.01, 95% CI 1.37–2.96), cancer (OR 1.73, 95% CI 1.05–2.84), other somatic disease (OR 1.20, 95% CI 1.04–1.39) and older age (OR 1.07, 95% CI 1.04–1.10) in the multivariate analysis (Fig. 1). Less than 5 years duration of formal residency in Sweden was associated with decreased odds of antidepressant initiation (OR 0.76, 95% CI 0.63–0.92), compared with those who arrived > 10 years ago in Sweden (Fig. 1). Compared with those born in Former Yugoslavia, refugees born in Somalia (OR 0.70, 95% CI 0.49–0.99) and Iraq (OR 0.79, 95% CI 0.63–0.99) were less likely to initiate antidepressant use.

**Table 3:** Univariate Odds Ratios (ORs) and 95% Confidence Intervals (CI) of factors associated with antidepressant initiation separately among refugees and Swedish-born youth 16–25 years of age with common mental disorders, CMD (2006–2016, *N* = 3936 in each group) in Sweden

	Refugees			Swedish-born		
	Non-initiator *N* = 2361	Initiator *N* = 1575	Unadjusted OR (95% CI)	Non-initiator *N* = 1617	Initiator *N* = 2319	Unadjusted OR (95% CI)
Age, mean (STD)	20.3 (2.8)	20.9 (2.7)	1.09 (1.06–1.11)	20.4 (2.6)	20.4 (2.6)	0.99 (0.97–1.02)
Female gender	50.9 (1202)	51.0 (803)	1.00 (0.88–1-13)	52.1 (842)	50.2 (1163)	1.08 (0.95–1.23)
CMD type						
Depression	16.5 (389)	43.9 (692)	1.00	13.9 (224)	37.0 (857)	1.00
Anxiety disorder	77.3 (1826)	49.6 (781)	0.24 (0.21–0.28)	79.7 (1288)	56.9 (1319)	0.27 (0.23–0.32)
PTSD	6.2 (146)	6.5 (102)	0.39 (0.30–0.52)	6.5 (105)	6.2 (143)	0.36 (0.27–0.48)
Type of living area						
Large city	42.2 (997)	42.1 (663)	1.00	43.4 (701)	41.4 (959)	1.00
Medium-sized municipality	36.9 (871)	36.2 (570)	0.98 (0.85–1.14)	36.3 (587)	36.8 (854)	1.06 (0.92–1.23)
Small municipality	20.9 (493)	21.7 (342)	1.04 (0.88–1.24)	20.4 (329)	21.8 (506)	1.12 (0.95–1.33)
Sickness absence						
≤ 90 days	2.2 (52)	5.9 (93)	2.80 (1.98–3.95)	5.9 (96)	7.2 (168)	1.24 (0.96–1.61)
> 90 days	0.7 (16)	1.0 (15)	1.47 (0.72–2.97)	1.3 (21)	1.3 (31)	1.04 (0.60–1.83)
Disability pension	1.7 (39)	2.4 (37)	1.43 (0.91–2.26)	2.5 (41)	2.2 (51)	0.86 (0.57–1.31)
Comorbidities						
Previous CMD	5.3 (126)	7.1 (111)	1.35 (1.03–1.75)	6.0 (97)	7.2 (168)	1.22 (0.95–1.59)
Substance abuse	4.0 (95)	5.0 (79)	1.26 (0.93–1.71)	6.5 (105)	7.3 (170)	1.14 (0.89–1.47)
Eating disorder	0.6 (13)	1.2 (19)	2.21 (1.09–4.48)	1.4 (23)	1.9 (45)	1.37 (0.83–2.28)
ADHD	1.0 (24)	1.0 (15)	0.94 (49–1.79)	6.9 (112)	5.5 (127)	0.78 (0.60–1.01)
Other mental and behavioral disorder	5.6 (133)	7.2 (114)	1.31 (1.01–1.69)	8.4 (135)	9.7 (225)	1.18 (0.94–1.48)
Previous suicide attempt	6.0 (141)	4.8 (76)	0.80 (0.60–1.06)	3.5 (57)	3.6 (84)	1.03 (0.73–1.45)
Cancer	1.5 (35)	2.8 (44)	1.91 (1.22–2.99)	3.2 (52)	3.2 (74)	0.99 (0.69–1.42)
Asthma	0.9 (20)	1.2 (19)	1.43 (0.76–2.69)	2.9 (47)	3.6 (84)	1.26 (0.87–1.81)
Other respiratory disease	7.1 (167)	7.2 (114)	1.03 (0.80–1.31)	7.8 (126)	7.9 (183)	1.01 (0.80–1.28)
Musculoskeletal disease	9.6 (227)	8.8 (139)	0.91 (0.73–1.14)	10.0 (161)	10.8 (251)	1.10 (0.89–1.35)
Other somatic disease	36.6 (863)	41.8 (659)	1.25 (1.10–1.42)	38.7 (626)	37.0 (857)	0.93 (0.81–1.06)
Other medication use						
Anxiolytics	4.7 (110)	13.4 (211)	3.17 (2.49–4.02)	4.9 (79)	14.9 (345)	3.40 (2.64–4.38)
Opioids	4.2 (99)	13.3 (210)	3.52 (2.74–4.51)	3.8 (61)	10.4 (242)	2.97 (2.23–3.97)
Hypnotics	2.5 (60)	5.8 (92)	2.38 (1.71–3.32)	3.2 (52)	4.7 (109)	1.48 (1.06–2.08)
Antipsychotics	1.0 (23)	2.2 (34)	2.24 (1.32–3.82)	0.8 (13)	1.9 (43)	2.33 (1.25–4.35)

*CMD* common mental health disorder, *PTSD* post-traumatic stress disorder

In the multivariate analysis, the factors associated with antidepressant initiation among Swedish-born individuals included previous use of anxiolytics (OR 3.11, 95% CI 2.39–4.05) and hypnotics (OR 2.42, 95% CI 1.77–3.31), and living in medium-sized (OR 1.18, 95% CI 1.02–1.38) or small municipality (OR 1.21, 95% CI 1.01–1.44) compared with large city (Fig. 2). ADHD or other hyperkinetic disorders were associated with decreased odds of antidepressant initiation (OR 0.69, 95% CI 0.51–0.93).

## Discussion

In this study, we found that refugee youth were less likely than Swedish-born individuals of the same age to initiate antidepressant use after their first diagnosis of a common mental disorder. They also initiated other psychopharmacotherapy less often and there were differences in the medications prescribed. Factors associated with antidepressant initiation included previous use of anxiolytics and hypnotics in both cohorts, and less than 5 years duration of residency in Sweden among refugees.

To our knowledge, this is the first study assessing antidepressant initiation in refugee youth compared with Swedish-born individuals. This finding is in line with previous studies reporting a decreased prevalence of psychotropic use [6] or specifically antidepressant use [7, 8, 10] in adult migrant or refugee populations. Moreover, initiation of use among adults has been found to be less frequent among non-Western immigrants after a hospital-treated depression episode [7] and they seem to be less likely to follow their general practitioners’ recommendation regarding antidepressant initiation [10]. Our study adds to this knowledge by showing similar patterns for refugees with CMDs during adolescence and young adulthood.

Possible explanations for considerably lower antidepressant initiation rates after CMD diagnoses may be the refugees’ attitudes towards mental disorders, such as negative perception of illness [20, 21], a lack of knowledge about CMDs and their treatment [22], different preference of treatment choice [23], or beliefs about antidepressants or other psychopharmacotherapies [24]. On an individual level, perceived barriers for accepting treatment, such as different beliefs about medication and concerns about adverse effects, may be important, or alternatively, lack of facilitators, such as perceived health benefits related to pharmacotherapy and having insight on the need for treatment [22]. These facilitators represent a potential point to target intervention for promoting mental health interventions, such as psychotherapy or additional medication use if indicated among refugees. There may also be poor communication between health care providers and refugees [25], which may be related to inadequate language skills, and poor cultural competence, above and beyond language.

Another possibility explaining differences in antidepressant initiation between refugees and Swedish-born includes that refugee youth may not be able to afford to buy medication. We did not adjust for income as the study population included adolescents and young adults, part of whom are likely to live at home with their parents. In fact, over half of Swedish-born individuals and one third of the refugees lived with their parents, and for a large proportion of the refugees, the family situation was unknown. Still, low income is likely a partial explanation as a previous study from Denmark noticed that initiation of antidepressant use after hospital discharge due to a depressive episode was impacted by the level of income [7]. Our observation on longer dispensing lag among refugees, i.e., time lapse between prescription date and dispensing date, may reflect a lower income level. On the other hand, it may also be due to doubts regarding the effectiveness of medication for the symptoms or fear of adverse effects.

In line with a previous Swedish study among adults [6], we found that newly resettled refugee youths (≤ 5 years) were less likely to initiate antidepressants than their counterparts who resettled over 10 years ago. Differences in the initiation between country or region of origin were mostly non-significant. A previous study from US among Asian Americans found that a < 10 years’ length of stay in the U.S. was associated with stigmatizing beliefs about depression, e.g., thinking that depression is a personal weakness, compared with those who had stayed at least 10 years [21]. Another possible explanation for not initiating antidepressants in the first years of resettlement is the so called “healthy migrant effect” (i.e., that migrants have or report better health status than natives). It is possible that this effect gets weaker with time spent in the host country [26]. This might be related to an increased prevalence of mental health problems necessitating pharmacological treatment over time. These temporal trends might be influenced by e.g., discrimination and problems of acculturation.

Moreover, a longer stay may also be related to a better knowledge on how the health-care system is organized and where to seek help in the new host country. Better knowledge and acceptance of the health care system and psychiatric medication might also explain the findings that previous prescription of anxiolytics/hypnotics, comorbid cancer as well as previous sickness absence ≤ 90 days were associated with increased odds for antidepressant initiation. Moreover, previous use of anxiolytics and hypnotics may describe a general polypharmacy of psychiatric medication, which is becoming more common among children and adolescents in Sweden [27], or that those persons are more likely to accept any pharmacotherapy for CMD. In general, any psychotropic drug increases also the likelihood of initiation of another psychotropic drug [28]. Still, the mentioned clinical factors for higher odds of antidepressant initiation might also reflect a higher medical severity of the patients with CMDs.

Refugees were less likely to initiate use of SSRIs than Swedish-born, despite the evidence that SSRIs are efficacious in treating major depressive disorders in children and adolescents with major depressive disorder; whereas other types of newer antidepressants either lack efficacy or would require more studies [18]. In fact, only fluoxetine was significantly more effective than placebo in the meta-analyses assessing evidence on the specific antidepressants among children and adolescents [18]. Contrary to this evidence, fluoxetine was not most often used antidepressant in our study but the second most common antidepressant among Swedish-born and only the fourth most common among refugees. This is also against international and Swedish clinical care guidelines recommending SSRIs as the first choice and stating that fluoxetine should be the first-line pharmacological treatment for children and adolescents with major depressive disorders [29, 30]. There are multiple possible reasons why refugees were less likely prescribed first-line pharmacotherapies, including differences in symptomatology as well as patient and prescriber preferences on acceptability of treatments. These reasons should be carefully examined in future studies.

Mirtazapine was significantly more common first antidepressant among refugees, which is not in line with Swedish recommendation indicating mirtazapine mainly for the treatment of treatment resistant depression. It is, however, possible that mirtazapine use reflects the treatment of sleep problems as low dose mirtazapine in general has some efficacy for insomnia [31] and Swedish guidelines mention mirtazapine as a possible option for anxiety and sleep problems among children and adolescents [30].

Other psychopharmacotherapies also differed between refugees and natives, with hypnotics being more commonly initiated among refugees and anxiolytics among Swedish-born. It is possible that CMDs more commonly present as severe sleep problems among young refugees, or they may be more willing to accept medication, which is targeted to sleep problems over other symptoms. Hydroxyzine was the most common other psychopharmacotherapy, which is against Swedish guidelines on treatment of depressive and anxiety disorders in children and adolescents, stating clearly that hydroxyzine use is not recommended [30]. Lack of efficacy or lacking evidence regarding it’s effect is also mentioned in many practice care guidelines [32, 33].

## Strengths and limitations

The major strengths of this study include the high quality and completeness of register data allowing individual information on a considerable number of covariates over a long time period. This includes the advantage of practically no loss to follow-up. Sweden is worldwide the only country with both available registers and relatively large populations on young refugees, allowing subgroup analyses and investigation of a number of potential risk factors. There are also limitations. The Prescribed Drug Register includes only information on prescriptions, which have been dispensed from a pharmacy, but lacks information on prescriptions, which have not been dispensed. For this reason, the study cannot analyse the prescription frequency differences between refugees and their Swedish-born counterparts. A recent study reported that the prevalence of antidepressant use among children and adolescents is increasing in Sweden [27], which shows that overall utilization trend is increasing. We also lack data on other treatments, e.g., psychotherapy, and thus, it is possible that refugee youth were more likely referred to psychotherapy. Lastly, common mental disorders were measured by specialized health care use, which reflects more severe cases as milder cases are typically handled in primary care. Refugees are known to use specialized health care less frequently than individuals born in the respective host country [11], which might suggest a higher medical severity in refugees than Swedish-born with CMDs in this study. In this regard, it is even more notable that young refugees with CMDs had lower antidepressant initiation rates.

## Conclusions

Among adolescents and young adult refugees with common mental disorders, the initiation of antidepressants is considerably lower compared to their Swedish-born peers. Refugees were also less likely to initiate SSRI use, although that is most often the recommended first-line pharmacotherapy in clinical care guidelines. Further studies should be conducted to assess underlying reasons for these differences. Health literacy programs and training in transcultural medicine for health care staff can be recommended to overcome these differences.

## Electronic supplementary material

Below is the link to the electronic supplementary material.

Supplementary file1 (DOCX 81 kb)
