# Supplementary material for: Initiation of antidepressant use among refugee and Swedish-born youth after diagnosis of a common mental disorder: findings from the REMAIN study
Source: Soc Psychiatry Psychiatr Epidemiol. 2020 Sep 10;56(3):463–74. doi: 10.1007/s00127-020-01951-4 (PMC7904723; doi:10.1007/s00127-020-01951-4)
Supplement: Supplementary file 1 — Supplementary file1 (DOCX 81 kb) [file 127_2020_1951_MOESM1_ESM.docx]

**Supplementary Figure 1**. Formation of study cohorts of individuals 16-25 years of age with a diagnosis of a common mental disorder (CMD) in specialized health care 2006-2016 in Sweden.


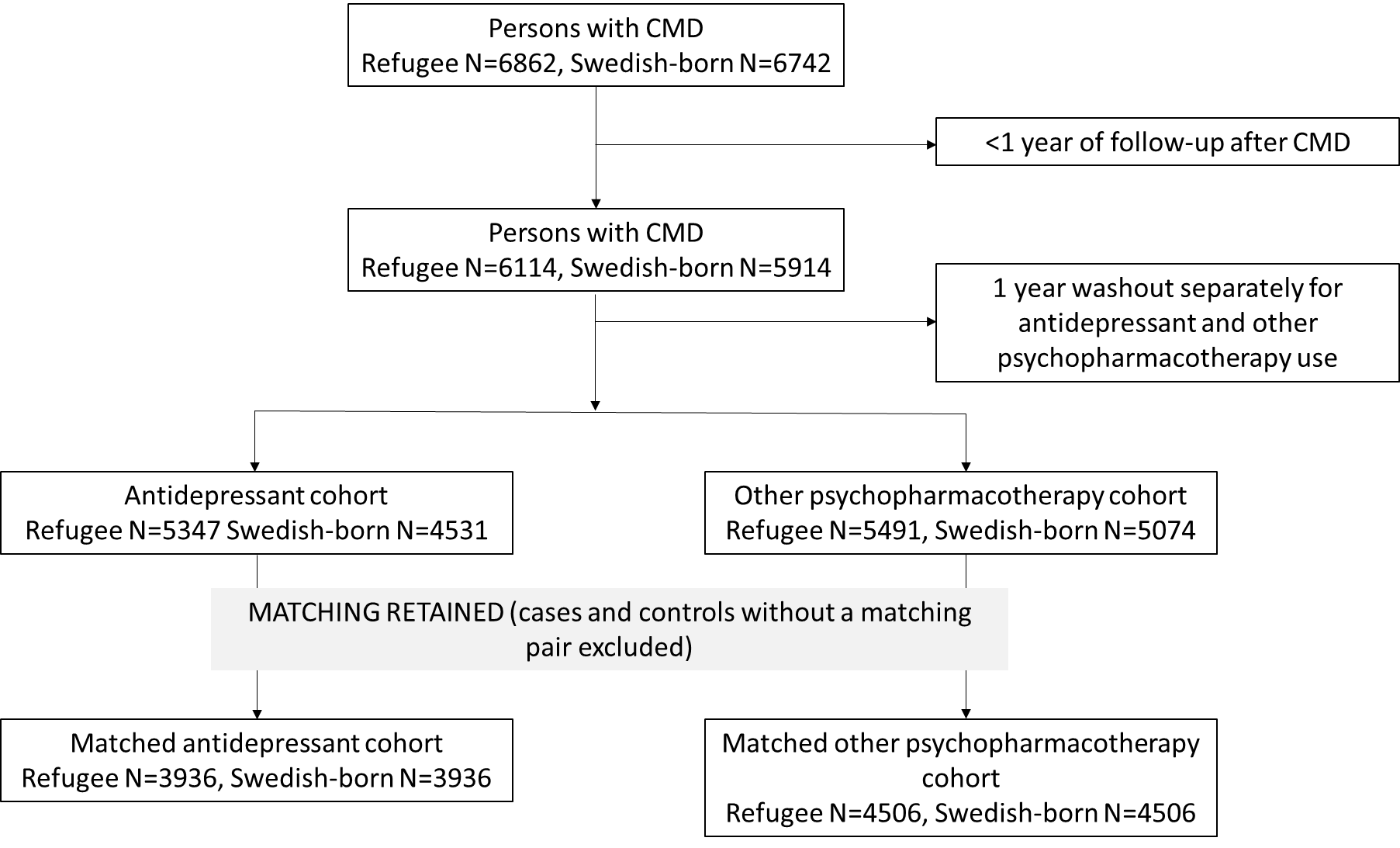


| **Supplementary Table 1.** Characteristics of refugees and matched Swedish born individuals 16-25 years of age with common mental disorders, CMD (2006-2016, n=3936 in each group) in Sweden. | | | |
| --- | --- | --- | --- |
|  | Swedish-born, % (n) | Refugees, % (n) | p-value |
| Female gender | 50.9 (2005) | 50.9 (2005) | matched |
| Mean age (SD) | 20.4 (2.6) | 20.5 (2.8) |  |
|  |  |  |  |
| **CMD type** |  |  | matched |
| Depression | 27.5 (1081) | 27.5 (1081) |  |
| Anxiety disorder | 66.2 (2607) | 66.2 (2607) |  |
| PTSD | 6.3 (248) | 6.3 (248) |  |
|  |  |  |  |
| **Type of living area*** |  |  | matched |
| Small municipality | 42.2 (1660) | 42.2 (1660) |  |
| Medium-sized municipality | 36.6 (1441) | 36.6 (1441) |  |
| Large city | 21.2 (835) | 21.2 (835) |  |
|  |  |  |  |
| **Attained education level*** | |  | <0.0001 |
| Low (≤9 years) | 47. 9 (1887) | 38.0 (1496) |  |
| Medium (10-12 years) | 33.4 (1316) | 22.5 (886) |  |
| High (≥13 years) | 9.3 (365) | 6.6 (259) |  |
| Missing | 9.4 (368) | 32.9 (1295) |  |
|  |  |  |  |
| **Family situation*** |  |  | <0.0001 |
| Cohabiting, living without children | 0.4 (14) | 2.3 (90) |  |
| Cohabiting, living with children | 2.0 (80) | 3.9 (154) |  |
| Single, living without children | 40.6 (1597) | 47.5 (1871) |  |
| Single, living with children | 1.1 (43) | 2.1 (81) |  |
| ≤20 years, living with parent/-s | 53.9 (2120) | 31.8 (1253) |  |
| Missing information | 2.1 (82) | 12.4 (487) |  |
|  |  |  |  |
| **Work disability***  Sickness absence |  |  | <0.0001 |
| ≤90 days | 6.7 (264) | 3.7 (145) |  |
| >90 days | 1.3 (52) | 0.8 (31) |  |
| Disability pension | 2.3 (92) | 1.9 (76) | 0.2121 |
|  |  |  |  |
| **Previous diagnoses (during 3 years before index CMD diagnosis)** | | | |
| Any mental and behavioral disorder | 20.5 (806) | 15.0 (591) | <0.0001 |
| Previous CMD | 6.7 (265) | 6.0 (237) | 0.1965 |
| Substance abuse | 7.0 (275) | 4.4 (174) | <0.0001 |
| Eating disorder | 1.7 (68) | 0.8 (32) | 0.0003 |
| ADHD/other hyperactivity disorder | 6.1 (239) | 1.0 (39) | <0.0001 |
| Other mental and behavioral disorder | 9.2 (360) | 6.3 (247) | <0.0001 |
| Previous suicide attempt | 3.6 (141) | 5.5 (217) | <0.0001 |
| Previous psychiatric hospital admission | 5.6 (219) | 4.4 (173) | 0.0172 |
| Previous non-psychiatric hospitalization | 20.3 (797) | 24.2 (954) | <0.0001 |
| Cancer | 3.2 (126) | 2.0 (79) | 0.0009 |
| Asthma | 3.3 (131) | 1.0 (39) | <0.0001 |
| Other respiratory disease | 7.9 (309) | 7.1 (281) | 0.2307 |
| Musculoskeletal disorder | 10.5 (412) | 9.3 (366) | 0.0823 |
| Other somatic disease | 37.7 (1483) | 38.7 (1522) | 0.3656 |
| **Previous drug use** (<6 months before CMD diagnosis) | | | |
| Anxiolytics | 10.8 (424) | 8.2 (321) | <0.0001 |
| Hypnotics | 7.7 (303) | 7.9 (309) | 0.8006 |
| Opioids | 4.1 (161) | 3.9 (152) | 0.6037 |
| Antipsychotics | 1.4 (56) | 1.5 (57) | 0.9245 |
| CMD: common mental health disorder; PTSD: Post-traumatic stress disorder; ADHD: Attention deficit hyperactivity disorder; p-value derived from Chi^2^-test; * measured in the year preceding the index CMD diagnosis | | | |

| **Supplementary Table 2.** Univariate Odds Ratios (ORs) and 95% Confidence Intervals (CI) of migration-related factors associated with antidepressant initiation among refugee youth 16-25 years of age with common mental disorders, CMD (2006-2016, n=3936) in Sweden. | | | |
| --- | --- | --- | --- |
|  | **Non-initiator**  **N=2361** | **Initiator**  **N=1575** | **Unadjusted OR**  **(95% CI)** |
| **Duration of formal residency in Sweden** |  |  |  |
| ≤5 years | 48.3 (1140) | 39.1 (616) | 0.66 (0.57-0.76) |
| 6-10 years | 16.0 (378) | 17.1 (270) | 0.87 (0.73-1.05) |
| >10 years | 35.7 (843) | 43.8 (689) | 1.00 |
| **Region of birth** |  |  |  |
| Former Yugoslavia | 19.8 (468) | 23.5 (370) | 1.00 |
| Somalia | 6.9 (162) | 4.3 (67) | 0.52 (0.38-0.72) |
| Other Africa | 6.3 (148) | 4.8 (75) | 0.64 (0.47-0.87) |
| Iraq | 23.0 (543) | 20.4 (322) | 0.75 (0.62-0.91) |
| Iran | 6.6 (155) | 8.7 (137) | 1.12 (0.86-1.46) |
| Syria | 3.5 (83) | 3.6 (56) | 0.85 (0.59-1.23) |
| Afghanistan | 17.9 (423) | 18.9 (297) | 0.89 (0.73-1.09) |
| Other Asia | 11.7 (275) | 10.9 (171) | 0.79 (0.62-0.99) |
| Chile/ South America | 2.1 (50) | 2.4 (38) | 0.96 (0.62-1.50) |
| Other/ unknown | 2.3 (54) | 2.7 (42) | 0.98 (0.64-1.51) |
